# Association between stress and lower urinary tract symptoms in children and adolescents

**DOI:** 10.1590/S1677-5538.IBJU.2019.0128

**Published:** 2019-12-17

**Authors:** Ana Aparecida Nascimento Martinelli Braga, Maria Luiza Teixeira Veiga, Maria Gabrielle Correia da Silva Ferreira, Hellen Maciel Santana, Ubirajara Barroso

**Affiliations:** 1 Centro de Distúrbios Urinários em Crianças (CEDIMI), Escola Bahiana de Medicina e Universidade Federal da Bahia, Salvador, Bahia, Brasil

**Keywords:** Urinary Bladder, Neurogenic, Stress Disorders, Traumatic, Adolescent

## Abstract

**Introduction:**

Lower urinary tract dysfunction (LUTD) is a common clinical condition. Emotional and behavioral issues are increasing among children and adolescents, with stress indicating difficulties in personal and social functioning. This study evaluated whether urinary tract symptoms (LUTS) is associated with stress.

**Materials and Methods:**

A cross-sectional, analytical study with 6-14-year-old patients with LUTS and no anatomical/neurogenic urinary tract abnormalities was conducted using the Dysfunctional Voiding Scoring System, a psychological assessment and the Child Stress Scale. The overall stress score was analyzed in relation to the psychological assessment data. Answers to the seven specific DVSS urinary questions were compared with those for the four Child Stress Scale domains. Univariate and multivariate analyses were performed. The chi-square test and Pearson's correlation were used to determine associations. Significance was defined as p <0.05.

**Results:**

Most children were male (56%). Mean age was 9.0±2.25 years. Stress was detected in 20 out of 98 patients (20.4%; 95% CI: 13-30%). Of these, 90% were born from unplanned pregnancies and 67% were upset about their disorder. All the Child Stress Scale domains were significantly associated with urinary dysfunction, with dysuria being significantly associated with all four domains. In the multivariate analysis, dysuria was the only symptom that remained associated with stress. Associations with stress strengthened as the frequency of dysuria increased: physical reactions (p <0.01), emotional reactions (p <0.05), psychological reactions with a depressive component (p <0.01) and psychophysiological reactions (p <0.05).

**Conclusion:**

Stress levels are higher in children and adolescents with LUTS who have more severe symptoms. Dysuria was the symptom most associated with stress, both in the physical reactions domain, in the psychological reactions domains with or without a depressive component and in the psychophysiological reactions domain.

## INTRODUCTION

Children and adolescents with lower urinary tract symptoms (LUTS) tend to have more emotional and behavioral abnormalities compared to those without this condition (1, 2–4). Individuals with LUTS may tend to be more anxious, to have difficulty concentrating and may be more prone to depression (5).

Stress is defined as our body's reaction to irritating situations or to those situations that make us extremely happy, fearful, excited or confused (6–8). Both physical and psychological components are involved in stress reactions. When this reaction functions as a motivator, it is referred to as positive stress or eustress; however, when out of control it is referred to as distress and becomes harmful (9). Stress negatively affects human health as a result of psychophysiological changes that occur when an individual is faced with a challenging situation. In children and adolescents, stress is commonly overlooked, while its principal element lies in the need to know how to deal with a new situation, whether good or bad (9, 10). Individuals with emotional issues may perceive their physical problems as extremely distressing and may feel less able to deal with them (11).

Children and adolescents with urinary symptoms are often punished, either verbally with no physical contact (reprimanded), physically without actual physical contact (e.g. being grounded by parents/caregivers) or even physically with actual physical contact (12, 13). Punishment is often a consequence of parents or caregivers tending to believe that it is intentional and failing to understand that urinary symptoms are an issue that merits careful attention, patience and understanding. The negative effects of urinary problems on the family, social relationships, academic performance and the quality of life of children and adolescents add to the seriousness of this detrimental condition (14).

Although studies have reported associations between emotional issues and urinary dysfunction, to the best of our knowledge no studies have dealt specifically with stress and its possible association with LUTS. A hypothesis was raised that children and adolescents with LUTS, with or without constipation, would experience different challenging situations. Hence, the findings of the present study could provide further, more objective information on this association for patients, their families and the professionals working in this area, providing better means of prevention and treatment and improving community health. Therefore, the objectives of this study were to test the hypothesis that children and adolescents with different lower urinary tract symptoms experience different degrees of stress, to describe the prevalence of stress in children and adolescents with LUTS, and to identify the lower urinary tract symptoms that are most associated with stress.

## MATERIALS AND METHODS

This was a cross-sectional analytical study conducted with children and adolescents receiving care at a pediatric referral facility for the interdisciplinary treatment of LUTS, with colleted data prospectively. Children of 6 to 14 years of age with lower urinary tract symptoms identified according to the Dysfunctional Voiding Scoring System (DVSS) (15), and no anatomical or neurological abnormalities that could explain these symptoms, were included in the study. Patients who did not complete the questionnaires or who refused to sign the informed consent form were excluded from the study.

All the patients were previously evaluated by a specialist, who also applied the DVSS. Next, a psychologist performed a psychological assessment of all the participants and applied the Child Stress Scale (6). The answers to the Child Stress Scale were stratified according to its four domains: physical reactions, psychological reactions, psychological reactions with a depressive component and psychophysiological reactions. This stratification is based on analysis of the content of the items grouped together into each of the domains. For instance, the items related to physical reactions deal with questions specifically referring to the body such as stomachache, sleep disturbances and urge to vomit, while those related to psychological aspects deal with worry, nervousness, sadness and fear. The domain of psychological reactions with a depressive component includes lack of energy, low self-esteem and a desire to disappear from the world. Items referring to feelings of embarrassment, eating-related anxiety, tachycardia and attention difficulties are part of the psychophysiological reactions domain.

The Child Stress Scale contains 35 questions, 9 in each of the first three domains and 8 in the psychophysiological reactions domain. There are five options of answers for each item, resulting in scores that range from 1 to 5 for the following answers, respectively: never, rarely, sometimes, often and always, based on the patient's recent past. The score for each of the domains is added together to obtain the individual's overall score for stress. Several criteria are deemed to determine the presence of stress: if the overall score is >105 points and/or if at least 7 items have been given the maximum score in each domain and/or based on a score ≥27 points for the first three domains and ≥24 points for the psychophysiological reactions domain.

Next, the answers to the DVSS were scored and compared with the overall score and the scores for each one of the four domains of the Child Stress Scale. The mean scores for each of the domains were added together to obtain the mean overall score for stress, which was used to describe the prevalence of the condition.

The association between the different symptoms of urinary dysfunction and stress was evaluated using the Statistical Package for the Social Sciences (SPSS), version 14.0 for Windows. Univariate and multivariate analyses were conducted, and significance was established at p <0.05. Due to the normality of the descriptive variables, means and standard deviations (SD) were used for the numerical variables and percentages for the categorical variables. The chi-square test was used to test the associations between the sociodemographic and clinical data and the overall score for stress (categorical data). Pearson's correlation was used to test the association between each of the symptoms of urinary dysfunction and the Child Stress Scale domains (numerical data). For the variables for which a statistically significant association was found, multiple linear regression was performed using the Enter method to insert the variables.

This study was conducted in accordance with the regulations and guidelines established in Resolution 466/12, which regulates the ethical issues associated with research involving human beings in Brazil. The institute's internal review board approved the study protocol under reference number 1.235.650. The children's parents or guardians signed an informed consent form and the children signed an informed assent form.

## RESULTS

The study included 98 children and adolescents. Of these, 55 (56%) were male. The age of the participants ranged from 6 to 14 years, with a mean of 9.0±2.25 years (Table-1).

**Table 1:** Characteristics of the children and adolescents participating in the study.

Characteristics			
		n	Mean±SD
Age (years)		98	9.0±2.25
		n	%
**Sex (n=98)**			
	Male	55	56.1
	Female	43	43.9
**Child's literacy (n=89)**			
	Literate	80	89.9
	Illiterate	9	9.2
**City of residence (n=91)**			
	Salvador	65	71.4
	Other	26	28.6
**Accompanying person (n=90)**			
	Mother	68	75.6
	Other	22	24.4
**Mother and father live together (n=87)**			
	Yes	59	67.8
	No	28	32.2
**Only child (n=88)**			
	Yes	16	18.2
	No	72	81.8
**Planned pregnancy (n=86)**			
	Yes	28	32.6
	No	58	67.4
**Mother attended prenatal care (n=86)**			
	Yes	85	98.8
	No	1	1.2
**Acceptance of the pregnancy (n=87)**			
	Yes	81	93.1
	No	6	6.1
**Attitude of the caregiver in relation to the voiding problems (n=81)**			
	Understanding	46	56.8
	Impatient	35	43.2
**Was the child/adolescent punished because of the voiding problems? (n=70)**			
	Yes	30	42.9
	No	40	57.1
**Reaction of the child/adolescent in relation to the voiding problems (n=78)**			
	Upset	31	39.7
	Indifferent	47	60.3

Based on the overall Child Stress Scale score (Appendix), 20 patients (20.4%) were found to have stress (95% CI: 13-30%). The mean score for each domain was 8.61±5.52 for physical reactions, 12.68±7.25 for psychological reactions, 6.99±6.98 for psychological reactions with a depressive component, and 9.80±6.05 for psychophysiological reactions.

Table-2 shows the association between characteristics of the participants obtained from the psychological assessment and the overall Child Stress Scale score. These results show that 90% of the patients with stress were born from an unplanned pregnancy. In addition, 67% were upset by their urinary disorder, suggesting an association between personal emotional issues, family relationships, and stress.

**Table 2:** Associations between the characteristics of the children/adolescents and the results of the Child Stress Scale.

Factor		Absence of Stress		Presence of stress		p-value
		n	%	n	%	
**Age (years) (n=98)**						
	6-10	55	70.5	14	70	1.000
	11-14	23	29.5	6	30	
**Sex (n=98)**						
	Male	41	52.6	14	70	0.209
	Female	37	47.4	6	30	
**Child's literacy (n=89)**						
	Illiterate	6	8.5	3	16.7	0.379
	Literate	65	91.5	15	83.3	
**Parents live together (n=87)**						
	No	21	29.6	7	43.8	0.374
	Yes	50	70.4	9	56.3	
**The patient is an only child (n=88)**						
	No	59	(84.3)	13	72.2	0.303
	Yes	11	(15.7)	5	27.8	
**Planned pregnancy (n=86)**						
	No	42	61.8	16	88.9	0.045*
	Yes	26	38.2	2	11.1	
**The mother received prenatal care (n=86)**						
	No	0	0.0	1	5.3	0.221
	Yes	67	100	18	94.7	
**The caregiver is accepting of the child (n=87)**						
	No	5	7.2	1	5.6	1.000
	Yes	64	92.8	17	94.4	
**Caregiver's attitude in relation to the voiding problems (n=81)**						
	Impatient	30	46.2	5	31.3	0.400
	Understanding	35	53.8	11	68.8	
**Punishment because of the voiding problems (n=70)**						
	No	32	59.3	8	50	0.573
	Yes	22	40.7	8	50	
**Patient's reaction to the voiding problems (n=78)**						
	Indifferent	42	66.7	21	33.3	0.037*
	Upset	5	33.3	10	66.7	

* Chi-square test, statistically significant with p <0.05.

The distribution of the various urinary symptoms in the children and adolescents was correlated with the different Child Stress Scale domains, as shown in Table-3. In all the domains, there were associations with some of the items in the DVSS. Consequently, an analysis was then conducted to determine whether there was any association between the periodicity of each individual symptom and a higher stress score, with results showing that stress level increased as a function of the intensity of the lower urinary tract symptoms. Scores for the domains of physical reactions, psychological reactions, psychological reactions with a depressive component and also psychophysiological reactions to stress were all found to increase as a function of the frequency of dysuria (Table-3). Furthermore, the psychophysiological reactions to stress intensified as a function of how often the occurrence of urinary incontinence was associated with soaked underwear. Nevertheless, following multivariate analysis, the only variable found to reliably predict stress was dysuria (Table-4).

**Table 3:** Correlation between the items evaluated in the Dysfunctional Voiding Scoring System (DVSS) and the domains of the Child Stress Scale.

		Physical Reactions		Psychological Reactions		Psychological Reactions with a Depressive Component		Psychophysiological Reactions	
DVSS items	n	R	p-value	R	p-value	R	p-value	R	p-value
Having wet clothes/underwear during the day	98	0.105	0.305	0.035	0.735	0.104	0.308	0.168	0.098
Soaked underwear during the day	98	0.147	0.150	0.051	0.621	0.198	0.051	0.213	0.035**
Voids only once or twice a day	98	0.001	0.996	0.054	0.597	0.020	0.844	0.000	0.997
Postpones voiding by crossing legs, squatting or dancing	98	0.219	0.030*	0.018	0.864	0.084	0.411	0.107	0.292
Can't wait when needing to void	98	0.068	0.503	0.072	0.482	0.062	0.542	0.044	0.669
Needs to strain to void	98	0.111	0.278	0.045	0.663	0.115	0.259	0.130	0.203
Painful voiding (in the preceding 30 days)	98	0.348	<0.01*	0.251	0.013**	0.319	p <0.01*	0.263	p <0.01*
Total score for the DVSS items 1, 2, 5, 6, 7, 8 and 9	98	0.227	0.025**	0.099	0.333	0.200	0.049**	0.221	0.029**

* Statistically significant with p <0.01;

** Statistically significant with p <0.05.

**Table 4:** Linear regression analysis between the items evaluated in the Dysfunctional Voiding Scoring System (DVSS) and the domains of the Child Stress Scale.

		Physical Reactions		Psychological Reactions		Psychological Reactions with a Depressive Component		Psychophysiological Reactions	
DVSS items	n	Beta	p-value	Beta	p-value	Beta	p-value	Beta	p-value
Soaked underwear during the day	98	0.096	0.450	0.014	0.916	0.172	0.185	0.163	0.214
Postpones voiding by crossing legs, squatting or dancing	98	0.206	0.135	-0.052	0.721	-0.019	0.890	-0.017	0.906
Painful voiding (in the preceding 30 days)	98	0.349	p <0.01*	0.237	0.026**	0.308	p <0.01*	0.246	0.018**
Total score for the DVSS items 1, 2, 5, 6, 7, 8 and 9	98	-0.047	0.782	0.080	0.658	0.043	0.805	0.081	0.649

* Statistically significant p <0.01;

** Statistically significant p <0.05.

## DISCUSSION

In the present study, stress levels were found to increase as a function of the frequency of some of the lower urinary tract symptoms. Not only was the presence of dysuria associated with stress but this association also became stronger as the frequency of this urinary complaint or the stress score increased. In other words, the more painful urination is, the greater the stress level associated with it. To the best of our knowledge, this is the first study to correlate LUTS with stress and these findings add a new spectrum of severity to this disorder.

LUTS have already been associated with emotional and behavioral problems (1, 2–4). Children with LUTS tend to be shyer, more irritable, and more prone to depression (5), or may display aggressive and transgressive behavior (4). Children diagnosed with attention deficit hyperactivity disorder and oppositional defiant disorder often experience urinary urgency and a reduced number of daily voids, respectively (4). These data may also show that brain function and its response to external stimuli are related to the urinary symptoms.

Stress is a state that results from exaggerated responses to external events interpreted as threats. This “exaggerated” response, which generates an atmosphere of anxiety and unsatisfactory emotional response, may perpetuate symptoms. A classic example is seen in cases of panic disorder and tachycardia. Anxiety develops as the result of some external event (threat), generating an adrenalin rush and tachycardia. The sensation generated by the increased heart rate results in further anxiety that, in turn, increases tachycardia, generating a feedback loop (16). Applying this to LUTS, such as in situations of overactive bladder, for example, urinary urgency (an event/threat) generates anxiety and brain hyperactivity, compounding the sensation of bladder filling (11), which, in turn, may increase stress levels.

Since dysuria was the symptom found to be most associated with increased stress, this merits particular consideration. The causes of dysuria can be manifold, including urinary infections, urethritis, nonbacterial inflammation of the bladder or urethra, and kidney stones (17, 18). There is no clinically demonstrable reason to believe that the patients evaluated in the present study would be included in any of these categories. The absence of an urinary infection or of any other clinical condition that could be responsible for lower urinary tract symptoms is a basic condition for a diagnosis of LUTS. Nevertheless, in clinical practice dysuria is often associated with poor hydration and an explosive voiding contraction resulting from significant urgency or bladder overdistension (19). The patients with dysuria may also be those with more severe lower urinary tract symptoms and, consequently, those with a poorer emotional response, less able to adapt to the events/threats they are forced to confront on a daily basis, hence generating stress. On the other hand, it is also possible that the symptom itself, by generating constant discomfort, could increase stress levels. The sensation of a full bladder provides an opportunity for an emotional response and the act of voiding is a behavior. It is unsurprising that an association was found between lower urinary tract symptoms and emotional and behavioral problems in children/adolescents with LUTS.

In current times, increased demands requiring immediate resolution may result in many children being unprepared to deal with this stress-generating process (8). In addition, adolescence involves a physical transition from childhood to adulthood, a moment when these individuals are no longer children but are not yet adults. This can be a stressful time, with teenagers feeling unprepared to deal with the new situations that present in their lives (9). Parents, who often insist on their children being independent, are the same parents who are often authoritarian and controlling, since they are also experimenting with this new role. Therefore, conflicts develop and the urinary symptoms, associated with emotional stress, may be indicative of a broader context that needs to be dealt with.

Around 20% of the patients evaluated were found to have stress. This is a higher percentage compared to the range of 10 to 15% previously reported in studies on emotional and behavioral disorders in general (3). Since there was no control group of individuals without LUTS in the present study, it is impossible to affirm that the rate found is higher than that of the general population. Nevertheless, this percentage can be useful as a reference in clinical practice and when providing information to parents. The figure of around 20% of patients with stress represents 20 children and adolescents found to be in a state of emotional fragility. The psychological reactions domain was that for which scores were higher, highlighting the need for greater attention in this area.

In the group of patients whose parents had not planned the pregnancy, the percentage of children/adolescents with stress was higher (90%), as was also the case for the group of individuals who stated that their urinary complaint upset them (67%), showing a link between personal emotional issues, family relationships, and stress.

There are some limitations to the present study. The lack of a control group prevented any comparison from being made between the stress levels found in this population of children and adolescents with LUTS and the general population of children and adolescents. In addition, the cross-sectional nature of the study precludes any conclusions from being reached with respect to cause and effect. Therefore, it remains unclear whether the stress is a consequence of LUTS or whether the emotional problems exacerbate the urinary symptoms. In addition, the sample size may have insufficient statistical power for the analysis of some of the variables.

Studies reporting on children and adolescents with urinary problems being punished (12, 13), on their social exclusion and on difficulties in family dynamics, impacting negatively on their quality of life and academic performance (14), are endorsed by the results of the present study. Our findings confirmed that an already existing source of distress (painful voiding) was associated with increased psychic suffering in the form of stress.

## CONCLUSIONS

Stress levels are higher in children and adolescents with LUTS who have more severe symptoms. Results show an overall prevalence of stress of 20%, with higher scores in the domain referring to the psychological reactions to stress. Dysuria was the symptom most associated with stress, both in the physical reactions domain, in the psychological reactions domains with or without a depressive component and in the psychophysiological reactions domain. These associations should be taken into consideration when managing the treatment of a child or adolescent with LUTS. Those reporting painful voiding need particular psychological care that will focus on possible personal and social issues and provide families with appropriate support and advice.

## APPENDIX

### Attachment: Child Stress Scale

#### Questionnaire

Authors: Marilda Emmanuel Novaes Lipp and Maria Diva Monteiro Lucarelli

**Table:**

Name:		
Date of Birth: / /	Place of birth: city/state/country:	
Age:	Sex: M ( ) F ( )	Schooling:
ID number:	Social security number:	
Class at school:	School/teaching institute: Public ( ) Private ( )	
Dominant hand: left ( ) right ( ) ambidextrous ( )		Profession:
Function:	Date of application: day/month/year	
Interviewer:	Starting time:	Finishing time:
I give my permission for this information to be used confidentially in research Signature:		
	
**Instructions:** The questions below deal with things that the children may have or feel. You should indicate to what extent the things described in each question happen by completing one of the drawings below.		

**Table:**

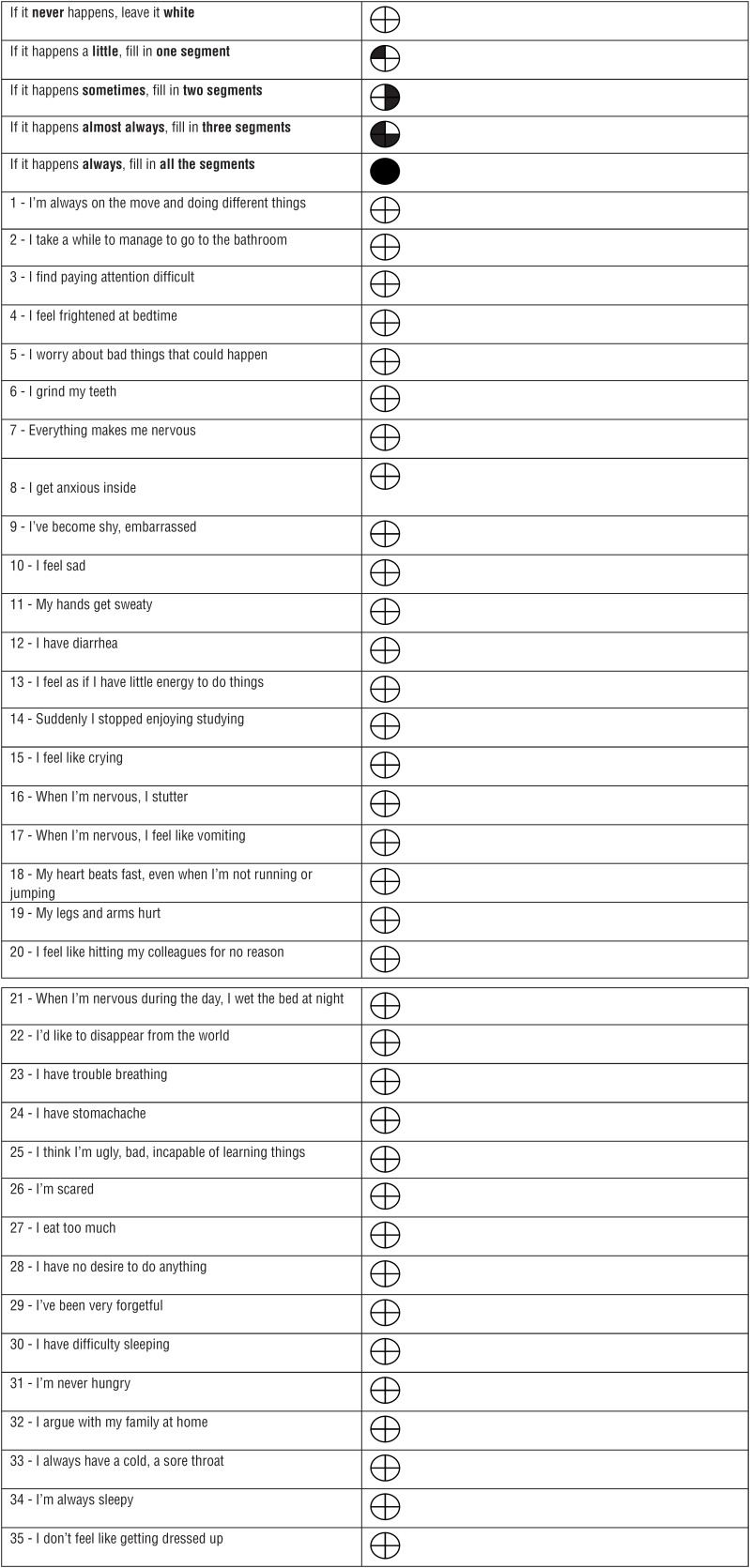

### Scoring sheet

Marilda Emmanuel Novaes Lipp/Maria Diva Monteiro Lucarelli

**Table:**

Name:		
Sex:	Age:	Education level:
School:		

**Table:**

Physical Reactions		Psychological Reactions		Psychological Reactions with a depressive component		Psychophysiological Reactions	
Items	Score	Items	Score	Items	Score	Items	Score
2		4		13		1	
6		5		14		3	
12		7		20		9	
15		8		22		16	
17		10		25		18	
19		11		28		23	
21		26		29		27	
24		30		32		33	
34		31		35			
**Total**							

### Conclusion

Attending psychologist:___________
